# The origin and spread of CRF85_BC, driven by heterosexual transmission among older people in Sichuan, China

**DOI:** 10.1186/s12879-020-05488-4

**Published:** 2020-10-19

**Authors:** Ling Su, Yi Feng, Shu Liang, Yali Zeng, Yiping Li, Hong Yang, Li Ye, Qiushi Wang, Dongbin Wei, Dan Yuan, Wenhong Lai, Linglin Zhang

**Affiliations:** 1 Sichuan Provincial Center for Disease Control and Prevention, Center for AIDS/STD Control and Prevention, Chengdu, 610041 Sichuan China; 2 Division of Virology and Immunology, National Center for AIDS/STD Control and Prevention (NCAIDS), China CDC, Beijing, China

**Keywords:** HIV-1, Recombinants, Heterosexually transmission

## Abstract

**Background:**

CRF_BC recombinants, including CRF07_BC and CRF08_BC, were considered the predominant subtypes in China. Since the discovery of HIV-1 circulating recombinant form CRF 85_BC in Southwest China in 2016, this BC recombinant forms had been reported in different regions of China. However, the history and magnitude of CRF85_BC transmission were still to be investigated.

**Method:**

We conducted the most recent molecular epidemiology of HIV-1 among newly reported HIV-1 infected patients in Sichuan in 2019 by sequencing and phylogenetic analysis of 1291 *pol* sequences. Then, we used maximum likelihood approach and the Bayesian Markov chain Monte Carlo (MCMC) sampling of *pol* sequences to reconstruct the phylogeographic and demographic dynamics of the CRF85_BC.

**Results:**

HIV-1 CRF85_BC (68/1291, 5.27%) became the fourth most prevalent strain revealing a significant increase in local population. CRF85_BC were only found in heterosexually infected individuals and the majority of CRF85_BC (95.45%) were circulating among the people living with HIV aged 50 years and over (PLHIV50+), suggesting a unique prevalent pattern. The founder lineages of CRF85_BC were likely to have first emerged in Yunnan, a province of Southwest China bordering Sichuan, in the early 2000s. It then spread exponentially to various places (including Guangxi, Sichuan, et al) and became endemic around 2008.6 (2006.7–2010.2) in Sichuan.

**Conclusion:**

Taken together, our findings on HIV-1 subtype CRF85_BC infections provided new insights into the spread of this virus and extended the understanding of the HIV epidemic in China.

## Background

The human immunodeficiency virus type 1 (HIV-1) epidemic remains a major public health burden in China. By the end of 2018, it is estimated that there are approximately 1.25 [1.10–1.40] million persons living with HIV/AIDS in China. During 2018, 81 [60–105] thousand new HIV infections occurred [1].

In early 1990s, HIV-1 Thai subtypes B′ and C were co-circulated among injection drug users (IDUs) [2] in Yunnan province in southwestern China bordering the ‘Golden Triangle’, leading to active inter-subtype recombination which generated CRF07_BC, CRF08_BC, and a large number of unique recombinant forms [3, 4]. In the initial period, drug trafficking activities were considered to be responsible for the rapid spread of CRF07_BC and CRF08_BC across China [5]. From 1978, as commercial sex activity increased across the country [6], the proportion of newly reported HIV infections/acquired immune deficiency syndrome (AIDS) patients through heterosexual transmission increased dramatically. Nowadays, CRF07_BC and CRF08_BC have become the most epidemic strains in China [7, 8] and drug-driven epidemic has been shifting to sexually transmission-driven epidemic [9]. There were other B/C CRFs reported in the last few years, such as CRF57_BC (identified in Baoshan, Western Yunnan) [10], CRF62_BC and CRF64_BC (identified in Ruili/Dehong, Western Yunnan) [11]. However, there was scarce evidence that such B/C recombinant forms had spread to other areas. In 2016, we reported a novel B/C recombinant form, CRF85_BC, among heterosexually infected patients in southern Sichuan (Yibin city) [12]. CRF85_BC was then reported in Anhui [13], Yunnan [14] and Northern Sichuan [15] (Guangyuan City). Due to lack of B or C epidemic history in the local high-risk population, the origin and the spread of CRF85_BC was still to be investigated.

Sichuan province is in the interior of Southwest China, adjacent to Yunnan and Guizhou in the South and Tibet in the West. Sichuan has a population of approximately 85 million people, and is a developing area in China. Since the initial HIV epidemic was fueled by IDU in 1990s, the HIV epidemic in Sichuan had become one of the worst in China. By 2018, there were 132, 680 HIV infections reported in Sichuan province, which ranked the first among all provinces/autonomous regions in China [16]. Several studies have focused on the HIV epidemic in IDUs [17, 18] and CRF07_BC [19, 20] in Liangshan prefecture in Sichuan, and reported that the infected IDUs in this region could serve as a source of transmission to other regions of China [20]. However, limited attention has been paid to the recent shift of the HIV-related risk behaviors from IDU to sexual contact, and also to other circulating HIV strains in this area.

Genetic and temporal dynamic analyses have been widely used to reconstruct the history of the HIV epidemic and have provided important information to aid in the development of strategies for the prevention of HIV-1 transmission. Here, we reported an investigation of the temporal and spatial dynamics of HIV CRF85_BC to give more clues about transmission of HIV strains in China.

## Methods

### Study population

A molecular epidemiological surveillance of Sichuan conducted among all newly HIV-1 diagnosed in April 2019. A total of 1573 antiretroviral-naive patient samples were collected at the first clinical visit. EDTA blood was collected with informed consent and 1291 HIV-1 *pol* sequences covering 1, 060 base pairs (HXB2: 2, 253–3, 312) were obtained as described previously [15]. Demographic data (age, gender & self-identified ethnicity) and relevant information associated with HIV-1 transmission were abstracted from the national surveillance database. This study was reviewed and approved by the ethics committees of Sichuan Center for Disease Prevention and Control.

### HIV-1 subtyping and pairwise distance analysis

HIV subtypes were assessed by phylogenetic analysis. The HIV-1 *pol* sequences were aligned with reference sequences of various subtypes and circulating recombinant forms (CRFs) from Los Alamos HIV sequence database (LANL, http://www.hiv.lanl.gov/). Multiple alignments were made automatically using the Mega version 7.0 [21] with minor manual adjustments. FastTree 2.3 was used to estimate an approximately-maximum likelihood phylogenetic tree for *pol* sequences using the GTR + G + I nucleotide substitution model [22]. The phylogenetic tree’s reliability was determined with local support values based on the Shimodaira-Hasegawa (SH) test [23] and presented using FigTree v1.4.3 (http://tree.bio.ed.ac.uk/software/figtree/). For sequences of unknown HIV-1 subtype, the jumping profile hidden Markov model (jpHMM) (http://jphmm.gobics.de/submission_hiv) was applied to screen for recombination breakpoints.

Pairwise distances of CRF85_BC sequences collected from two cross-sectional surveys (in 2014 and 2019) were computed using the Tamura-Nei model in the MEGA v7.0 software [21] .

### CRF85_BC sequences dataset

CRF85_BC HIV-1 *pol* sequences covering 1, 060 base pairs (HXB2: 2, 253–3, 312) were derived from multiple ways, form (i) molecular epidemiological surveillance of Sichuan in 2019(*N* = 68), form (ii) molecular epidemiological surveillance of Sichuan in 2014 [24] (*N* = 14), from (iii) Drug resistance surveillance database in Sichuan CDC between 2012 and 2019 (*N* = 58), (iv) from other Molecular epidemiological investigation conducted in Sichuan [25] (*N* = 9), and (v) all other CRF85_BC (Yunnan: *n* = 2; CQ: *n* = 1) and the sequences (Yunnan: *n* = 7; Anhui: *n* = 2; Guangxi: *n* = 2; HN: *n* = 1) that are most closely related to each of the Sichuan CRF85_BC sequences with known sampling dates by BLAST search in the Los Alamos HIV database (LANL, http://www.hiv.lanl.gov/). All the sequences were confirmed as CRF85_BC by phylogenetic analysis mentioned above.

To avoid biases due to convergent evolution introduced by drug resistance selection rather than genealogical similarity, known surveillance drug resistance mutations (SDRM) have been excluded from the sequences prior to any analyses as described previously [26, 27].

### Bayesian MCMC evolutionary analysis

Estimation of evolutionary rate and the time of the most recent common ancestor (tMRCA) for CRF85_BC were performed by BEAST v1.8 [28]. A Bayesian skygrid model with Markov chain Monte Carlo (MCMC) inference under the relaxed lognormal molecular clock was selected as a reliable mode for this analysis. The MCMC chains were run 200 million times with 20,000 generations logged in, and the first 10% generations were discarded as burn-in. Bayesian MCMC output was analyzed using TRACER v1.5 [29], and all parameters were estimated from an ESS > 200. The trees were summarized in a target tree using the Tree Annotator program and scanned using the Fig. Tree 1.4.3.

### Statistical analysis

Chi-square test was used to compare the distributions of the population and demographic information among various HIV-1 strains. *P* values less than 0.05 were considered as statistical significance. All statistical analyses were performed using SPSS v.20.0 software (IBM Company, New York, USA).

## Results

### Molecular epidemiological surveillance of Sichuan in 2019

**Fig. 1:**
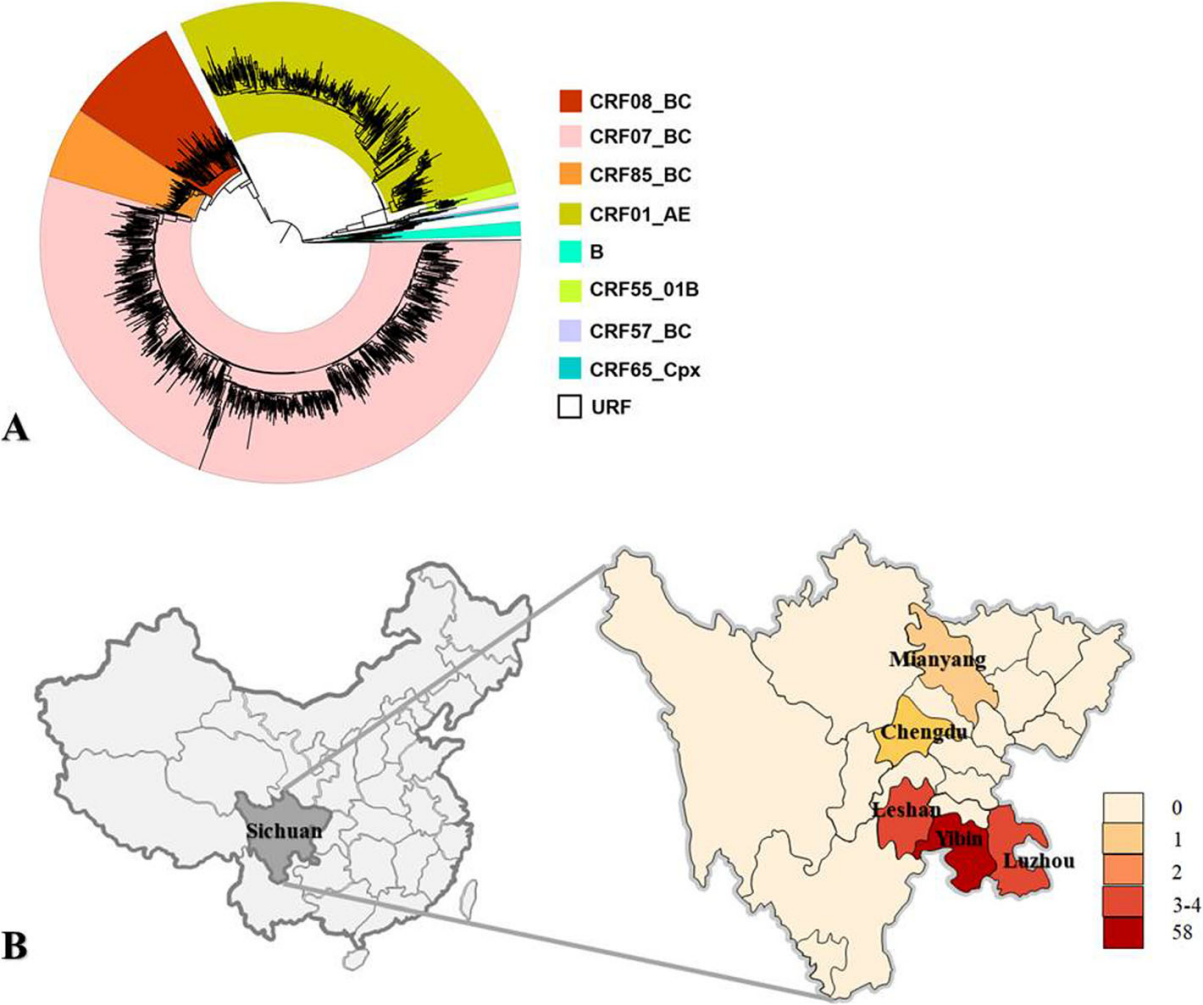
The distribution of CRF85_BC from Sichuan in 2019. **a** Phylogenetic analysis of *pol* sequences from Sichuan in 2019. The phylogenetic tree was constructed using the approximately-maximum-likelihood method based on the *pol* region (HXB2: 2, 253 to 3, 312 nt) in FastTree 2.3. The nucleotide substitution mode was GTR + G + I. The subtypes and circulating recombinant forms are marked in different colors. The bootstrap value is indicated at relevant nodes. HIV-1 group O was chosen as an out-group in the rooted tree. The reference sequences from the Los Alamos HIV sequence database (http://hiv-web.lanl.gov/content/index). **b** The demographic distribution of CRF85_BC in Sichuan. Number of reported cases in different prefectures in Sichuan based on the molecular epidemiological surveillance of Sichuan in 2019

Of the 1291 available sequences in the molecular epidemiological surveillance of Sichuan in 2019, CRF07_BC (676/1291, 52.36%), CRF01_AE (341/1291, 26.41%), CRF08_BC (114/1291, 8.83%), and CRF85_BC (68/1291, 5.27%) were the 4 most epidemic strains (Fig. 1a). CRF85_BC dominated the HIV-1 epidemics in Yibin (58/146), a southeast city in Sichuan Province, and was also detected in Luzhou (4/174), Leshan (3/120), Chengdu (2/312) and Mianyang (1/70) (Fig. 1b). Compare with the data of Sichuan molecular epidemiological surveillance in 2014 [24], the proportion of CRF85_BC in Sichuan increased from 3.66% (14/383) to 5.27%, and it became more widely distributed. To further understand whether CRF85_BC was involved in ongoing transmission, we examined the sequence diversity by estimating pairwise distances between sequences in 2014(*n* = 14) and sequences in 2019(*n* = 68). We detected a much more diversity in 2019 (mean distance 0.023, SD 0.002) compared with 2014 (mean distance 0.010, SD 0.001), supporting an ongoing evolution of CRF85_BC overtime.

### A heterosexual transmission of CRF85_BC in Sichuan Province

The epidemiological and clinical features of the patients harboring CRF85_BC did not change significantly between 2014 and 2019 (Table 1). CRF85_BC were only found in heterosexually infected individuals, including commercial sexual transmission (64.7% in 2019 and 42.9% in 2014), non-marital and non-commercial sexual transmission (30.9% in 2019 and 21.4% in 2014) and spousal transmission (4.4% in 2019 and 35.7% in 2014). The majority of CRF85_BC, in 2014(78.6%) and 2019 (95.6%), was circulating among the people living with HIV aged 50 years and over (PLHIV50+), significantly higher than other HIV CRFs (*P* = 0.000, χ^2^ test). Patients with low education levels took a relatively larger proportion.

**Table 1:** Characteristics of patients harboring CRF85_BC in the molecular epidemiological surveillance of Sichuan conducted in 2014 and 2019

Characteristics	2014	2019
**Region[%; (n)]**		
Yibin	92.9 (13)	85.3 (58)
Panzhihua	7.1 (1)	─
Luzhou	─	5.9 (4)
Chengdu	─	2.9 (2)
Leshan	─	4.4 (3)
Mianyang	─	1.5 (1)
**Gender**	─	
Male	57.1 (8)	73.5 (50)
Female	42.9 (6)	26.5 (18)
**Age at diagnosis (year)**		
30–49	21.4 (3)	4.4 (3)
50–60	35.7 (5)	19.1 (13)
≥ 60	42.9 (6)	76.5 (52)
**Modality of transmission**		
Commercial Heteosexual	42.9 (6)	64.7 (44)
Non-marital and non-commercial Heteosexual	21.4 (3)	30.9 (21)
Spousal Heteosexual	35.7 (5)	4.4 (3)
**Education level**		
Primary school and below	78.6 (11)	75.0 (51)
Middle school	21.4 (3)	23.5 (16)
High school	─	1.5 (1)

### Estimated timeline and migration pathway of CRF85_BC

**Fig. 2:**
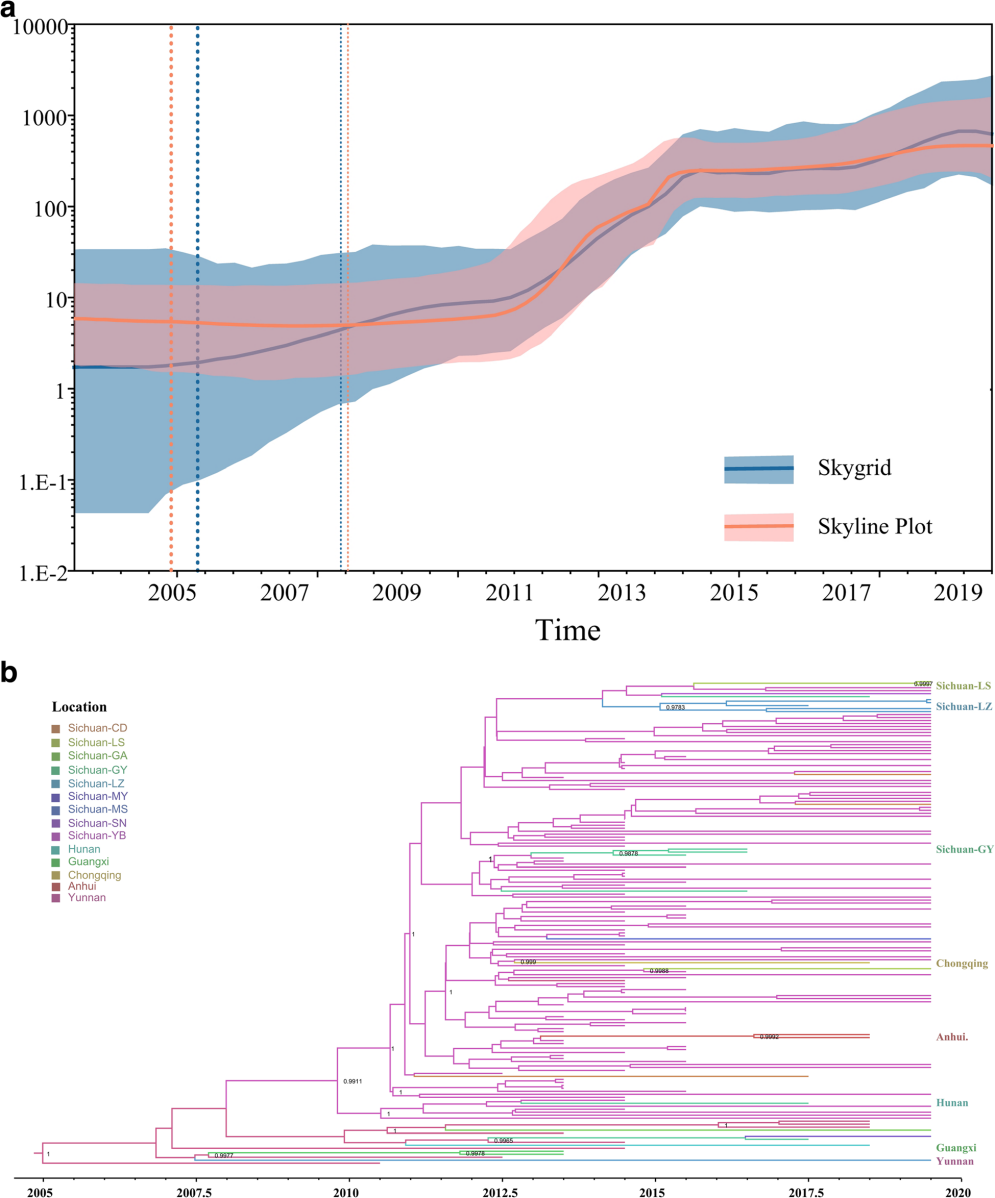
Prevalence and demographic history of CRF85_BC in Sichuan. **a** The Bayesian skyline plot and Bayesian skygrid analysis of CRF85_BC. The Bayesian skyline plot and Bayesian skygrid analysis estimated the past population dynamics of CRF85_BC. The thick solid line in the plot represents the median estimate, and the shaded region represents the 95% HPD credible region. **b** The MCC tree of CRFF85_BC. The MCC tree was constructed based a data set of 148 CRF85_BC pol region (HXB2: 2, 253 to 3, 312 nt) using Bayesian MCMC analysis implemented in BEAST v 1.8.4. The analysis was performed using an uncorrelated lognormal relaxed clock model in GTR + I + G4 nucleotide substitution model under an exponential coalescent model. The MCMC analysis was run for 200 million generations, with sampling every 20,000 generations. The different color branches indicate the strains from different areas, respectively. Sichuan-CD (Chengdu), Sichuan-LS (Leshan), Sichuan-GA (Guangan), Sichuan-GY (Guangyuan), Sichuan-LZ (Luzhou), Sichuan-MY (Mianyang), Sichuan-MS (Meishan), Sichuan-SN (Suining), Sichuan-YB (Yibin)

To elucidate the sources and dissemination routes of endemic CRF85_BC geographically, we constructed maximum clade credibility (MCC) tree based on 149 Sichuan sequences and 15 sequences from other provinces in China using Bayesian geographical evolution method. The corresponding median coefficient of rate variation was 0.33(95% highest posterior density (HPD): 0.18–0.49), supporting the selection of a relaxed molecular clock model. Both Bayesian skyline plot and Bayesian skygrid analysis showed that HIV-1 CRF85_BC experienced 2 fast growth phases during 2011–2014 and 2017–2019 (Fig. 2a) with a mean growth rate of 2.5*10^− 3^ per year [95% HPD: 2.0*10^− 3^ - 3.0*10^− 3^]. The tMRCA was inferred to be 2005.4 (95% HPD: 2002.3–2007.9), implying the origin time of the CRF85_BC (Table 2).

**Table 2:** Estimated dates of CRF85_BC origin in different areas

Regions		Year	*95%HPD*
Province	City		
Yunnan		2005.4	2002.3–2007.9
Guangxi		2012.3	2011.3–2013.2
Anhui		2017.9	2016.8–2018.5
Sichuan			
	Yibin	2008.6	2006.7–2010.2
	Luzhou	2014.3	2012.7–2015.8
	Leshan	2019.1	2018.2–2019.5
	Guangyuan	2013.6	2012.3–2015.0

The MCC tree showed that CRF85_BC from Yunnan were located at the root of the tree (posterior probability (PP = 1). Sequentially, virus spread form Yunnan to Sichuan (PP = 0.9911), Guangxi (PP = 0.9978), and other regions. The first place of the viral introduction into Sichuan was probably Yibin in 2008.6 (2006.7–2010.2). An explosive outbreak was then found in the heterosexual elderly people in this city. Several small clusters further suggested that the virus became epidemic in many cities of Sichuan (Luzhou, Guangyuan, et al.) and had rapidly spread to other provinces (such as Liaoning, Anhui and Chongqing) (Fig. 2b).

Notably, most Sichuan sequences originated from Yibin, except for 4 sequences. A Guangyuan and Mianyang sequence directly formed a sub-cluster with a sequence from Yunnan (PP = 0.9965) while a Luzhou strain grouped significantly (PP = 0.9977) with the sequences from Guangxi (Fig. 2b).

## Discussion

Consistent with the differences in viral diversity among geographical regions in China [8], over 66.5% circulating HIV strains in Sichuan were defined as CRF_BC including CRF07_BC, CRF08_BC and CRF85_BC et al. Hence, CRF_BC infections became a more serious public health issue, which necessitated a deeper understanding of the CRF_BC epidemic patterns in China. CRF85_BC, a newly identified CRF_BC, were the fourth most prevalent HIV strains (68/1291, 5.27%) in Sichuan and was dominating the HIV-1 epidemic in a southwestern city, i e. Yibin city.

In the present study, we only found CRF85_BC prevalent in heterosexually infected individuals. In molecular epidemiological surveillance of Sichuan in 2019, 64.7% of CRF85_BC were transmission via commercial sex. Other early studies about CRF07_BC and CRF08_BC suggested that CRF_BC had spread from IDU to the general population by sexual transmission [30]. Due to lack of the early CRF85_BC sequence, we could not rule out the possibility that this kind of CRF_BC originated from other high-risk groups such as IDUs or former plasma donors (FPDs) [13]. However, commercial sex transmission was at least an important driving factor of CRF85_BC becoming endemic in some local areas. Notably, the majority of cases infected by CRF85_BC were PLHIV50+ cases, most of whom were low-educated. PLHIV50+ have been significantly increasing in the past two decades globally [31]. The proportion of PLHIV50+ around the world increased substantially from 8% in 2000 to 16% in 2016 and is expected to reach an estimated 21% by 2020 [32]. Poor awareness about safer sex practices and underestimating their risk of contracting HIV made them extremely vulnerable to HIV.

Analysis of 148 PR/RT sequences from CRF85_BC infected individuals sampled between 2011 and 2019 revealed that this strain had not just become endemic in Sichuan but also spread to some other provinces in China. Until now, at least 6 provinces have been affected and had a most recent common ancestor (tMRCA) is estimated at around 2005. Yunnan is considered to be the origin area. Previous studies in Yunnan between 2009 and 2012 revealed several CRF_BC HIV-1 strains circulating among local sexually active youth, which is coincident with the gateway position of Yunnan in China’s HIV epidemic history [33]. By Bayesian skyline plot and Bayesian skygrid analysis, the exponential increase in the effective number of CRF85_BC indicated that the virus experienced 2 fast growth phases during 2011–2014 and 2017–2019.

CRF85_BC dispersion showed a strong geographic compartmentalization. After its origin in Yunnan, CRF85_BC almost at the same time spread to neighboring provinces such as Sichuan around 2008.6 (2006.7–2010.2), Guangxi around 2012.3 (2011.3–2013.2). The first place where the virus was introduced into Sichuan was probably Yibin city, which was located in the junction of Yunnan, Guizhou and Sichuan Provinces. In recent years, the local epidemic situation had deteriorated rapidly [34]. The explosive epidemic of CRF85_BC was found in the heterosexual elderly people in this city, and 39.7% (58/146) HIV cases locally were infected by CRF85_BC. Phylogeographical reconstruction further suggested that CRF85_BC spread from Yibin to other cities of Sichuan and even other provinces. This virus was identified in 9 cities in Sichuan, 3 of which probably had an endemic, such Luzhou, Leshan and Guangyuan. Leshan and Luzhou were both bordering with Yibin. Guangyuan is a northeastern city in Sichuan. According to another research, CRF85_BC infected people with Yibin household registration travelled to and resided in Guangyuan, further adding fuel to local endemic.

CRF85_BC entered Sichuan probably through multiple introduction events. In phylogenetic trees, some single strains of Sichuan also formed sub-clusters with Yunnan or Guangxi sequences. However, not all imported cases of Sichuan caused local epidemics.

## Conclusions

Our study is the first report to clarify the phylogeographic and demographic dynamics of CRF 85_BC, a newly-identified and increasingly prevalent HIV-1 strain, in Sichuan, China. This study also highlights that heterosexual behavior among older people played an important role in the contribution of CRF 85_BC transmission. The origin of CRF 85_BC was traced back to Yunnan around 2005, spread exponentially to various places (including Guangxi, Sichuan, et al), and later on became endemic around 2008.5 (2007.0–2010.5) in Sichuan. Our findings provide novel insights into the dissemination of a novel HIV-1 virus and extend the understanding of the HIV epidemic in China.

## Data Availability

The CRF85_BC sequences determined in this study are available in GenBank under Accession Numbers MT522696 - MT522844. Other datasets used and/or analyzed during the current study available from the corresponding author on reasonable request.

## Abbreviations

PLHIV50 +: The people living with HIV aged 50 years and over
CRFs: Circulating recombinant forms
IDUs: Injecting drug users
LANL: Los Alamos National Laboratory
MCMC: Bayesian Markov chain Monte Carlo
SDRM: Surveillance drug resistance mutations (SDRM)
SH: Shimodaira-Hasegawa
tMRCA: The most recent common ancestor
HPD: Highest posterior density
PP: Posterior probability
